# Lower Activity and Function Scores Are Associated with a Higher Risk of Preoperative Deep Venous Thrombosis in Patients Undergoing Total Hip Arthroplasty

**DOI:** 10.3390/jcm9051257

**Published:** 2020-04-26

**Authors:** Toshiyuki Kawai, Koji Goto, Yutaka Kuroda, Shuichi Matsuda

**Affiliations:** Department of Orthopaedic Surgery, Kyoto University, Kyoto 606-8507, Japan

**Keywords:** deep venous thrombosis, total hip arthroplasty, University of California Los Angeles activity score, Oxford Hip Score, preoperative deep venous thrombosis

## Abstract

This study was performed to investigate the relationship between patients’ activity and function levels and the incidence of preoperative deep venous thrombosis (DVT) prior to total hip arthroplasty (THA). We retrospectively reviewed 500 patients admitted for primary or revision THA from July 2014 to October 2018. The diagnosis of DVT was confirmed using Doppler ultrasonography 1 month before THA. The patients’ activity and hip function were evaluated using several clinical scores: the Harris Hip Score (HHS), Oxford Hip Score (OHS), University of California Los Angeles (UCLA) activity score, and visual analog scale (VAS) score. Those scores and the medical history were examined for correlations with preoperative DVT using univariate and multivariate models. Univariate regression analysis showed that older age, current steroid use, anticoagulant use, a history of DVT, collagen disease, a lower UCLA activity score, and a lower OHS were associated with an elevated risk of preoperative DVT. The multivariate analyses showed that a higher UCLA activity score (odds ratio (OR): 0.0049–0.012) and higher OHS (OR: 0.0012–0.0088) were associated with a lower risk of preoperative DVT in each model. Age (OR: 1.07 in both models), current steroid use (OR: 9.32–10.45), and a history of DVT (OR: 27.15–74.98) were associated with a higher risk of preoperative DVT in both models. Older age, current steroid use, a history of DVT, a lower UCLA activity score, and a lower OHS were risk factors for preoperative DVT before THA, even when controlling for potential confounders. Patients exhibiting low activity and low function levels were more likely to have DVT, even before surgery.

## 1. Introduction

Deep venous thromboembolism (DVT) is still a common complication after hip arthroplasty and can lead to pulmonary thromboembolism (PTE), although recent reviews have shown a significant decrease in the incidence of fatal PTE after total hip arthroplasty (THA) [1,2]. According to the American College of Chest Physicians’ evidence-based clinical practice guideline, hip surgery itself is a risk factor for DVT [3]. If patients undergoing THA have DVT before the surgery, the risk of extension of the thrombus and fatal PTE could be higher. Because patients with hip disability tend to be less active, physicians should be aware of the possibility that patients might already have DVT, even before the arthroplasty. Only a few studies have investigated the incidence of and risk factors for preoperative DVT [4,5], and these studies showed that the prevalence of DVT prior to THA ranged from 5% to 12%. To avoid critical complications associated with significant mortality, it is essential to determine the risk factors for DVT in patients with hip disability undergoing THA. 

Several risk factors for DVT after THA have been reported, including age [6,7,8,9,10,11,12,13], female sex [7,8,9,10,11,13,14,15,16], a higher body mass index (BMI) [6,17], malignancy [6,7,11], and congestive heart disease [6,9,11,13]. Bed rest [18] and immobilization [19,20] are also potential risk factors for DVT; however, no studies have quantified the activity level and evaluated the effect of the activity level on the risk of DVT.

Several scoring tools are used to evaluate the activity of patients with hip disease. The Oxford Hip Score (OHS) is a patient-reported outcome measurement designed to evaluate hip function and pain. This score has been proven to be reliable and valid and has been used in many clinical trials since its introduction in 1996 [21,22]. The University of California Los Angeles (UCLA) activity score is another widely used clinical measurement tool. This score is derived from a 10-point activity scale that evaluates patient activity based on 10 descriptive activity levels, ranging from wholly inactive and dependent (level 1) to regular participation in impact sports, such as jogging or tennis (level 10) [23]. The UCLA activity score reportedly has the strongest correlation with other patient activity measurements, has the highest reliability, and is the most effective activity rating scale for patients undergoing total hip and knee arthroplasty [24]. The Harris Hip Score (HHS) is one of the most commonly used clinical-based outcome measures and consists of pain, function, and range-of-motion components. Because these measurements were designed for similar purposes, they show significant correlations (OHS and HHS [25,26], UCLA and HHS [24,27,28], and UCLA and OHS [24,29]).

This study was performed to determine whether clinical measurements of patient activity and function are associated with the risk of DVT prior to THA.

## 2. Patients and Methods

This was a retrospective study of primary and revision THAs performed from July 2014 to October 2018. During this period, patients undergoing primary or revision THA were subjected to preoperative ultrasound examination for the detection of DVT. All patients provided informed consent, and the study protocol was approved by the institutional review board of our hospital (#E884-2, approved on 19 May 2014). In total, 507 primary and revision THAs were performed at our institute during the study period. Exclusion criteria were acute trauma (fracture) and symptomatic DVT at the time of surgery. Three primary THAs performed for femoral neck fractures and four revision THAs performed for periprosthetic fractures were excluded from this study. There were no patients who had symptomatic DVT at the time of the index surgery.

Therefore, 500 THAs (425 primary THAs and 75 revision THAs) were included in the study. The reason for the index THA was secondary osteoarthritis (OA) due to developmental dysplasia in 249 cases, avascular necrosis of the femoral head in 82 cases, primary OA in 71 cases, rapidly destructive coxarthrosis in 12 cases, rheumatoid arthritis in 6 cases, post-traumatic OA in 4 cases, and OA secondary to infection in 2 cases. The reason for the index revision THA was aseptic loosening in 55 cases, infection in 9 cases, migration of the bipolar head into the acetabulum in 6 cases, and recurrent dislocation in 5 cases. Fifty-one patients were taking one or more anticoagulants for the treatment or prevention of myocardial infarction (13 cases), cerebral infarction (10 cases), atrial fibrillation (Af) (7 cases), arrhythmia other than Af (2 cases), valvular heart disease (4 cases), and unknown reasons (15 cases). 

For the evaluation of patient activity and hip function, preoperative clinical assessments were performed using the HHS, UCLA activity score, and OHS. The OHS was calculated according to the modified scoring system, in which each question was scored from 0 to 4, with 4 representing the best outcome or fewest symptoms. The overall score ranged from 0 to 48, with 48 being the best outcome. The patients rated the pain intensity of the affected hip joint using the VAS, which ranged from 0 (no pain) to 10 (worst imaginable pain). These clinical scores were obtained 1 month before THA. We considered the following patient-related covariates: age, sex, BMI, a history of major surgery, a history of major surgery within the last 12 months, a known history of DVT, current steroid use, administration of anticoagulants, smoking status, and the presence of comorbidities (congestive heart failure (CHF), Af, diabetes mellitus (DM), collagen disease, or malignancy). Major surgery was defined as surgery requiring anesthesia (general, orthopedic, neurologic, or gynecologic surgery), as reported in a previous study [30]. Before the arthroplasty, patients were asked by the surgeon and a nurse independently about their history of major surgery and the presence of comorbidities. All patients underwent ultrasonography approximately 1 month prior to THA. Experienced clinical technicians blind to the patients’ backgrounds and clinical measurements performed all examinations using Doppler ultrasonography (Aplio 300, 500, or i800; Canon Inc., Tokyo, Japan).

### Statistical Analysis

Differences in proportions were calculated by the chi-square test. Differences in means were calculated by the Wilcoxon test to compare two groups. Probability values of <0.05 were considered significant. In the univariate regression, the dependent variable was preoperative DVT and the independent variables were age, sex, BMI, current steroid use, current anticoagulant use, smoking status, a history of DVT, a history of major surgery, a history of major surgery in the last 12 months, the presence of comorbidities (Af, CHF, DM, collagen disease, or malignancy), and preoperative clinical measurements (HHS, OHS, UCLA activity score, and VAS score). To examine the presence of potential multicollinearity in the following multivariate logistic analysis, the correlation among clinical scores was tested using the Spearman rank correlation coefficient. 

In the multivariate logistic regression analyses, the dependent variable was preoperative DVT and the independent variables were demographic parameters and clinical measures reaching a significance level of *p* < 0.05 in the univariate analysis. To evaluate the presence of multicollinearity, the variance inflation factors for the selected variables were examined in each model. Receiver operating characteristic (ROC) curves were used to examine the relations between the true positive rate (sensitivity) and false positive rate (1 − specificity) and the areas under the ROC curve (AUC). The Youden index (sensitivity + specificity − 1) was used to determine the optimal predictive cutoffs for the clinical scores that showed significant relevance for the preoperative DVT risk.

All statistical analyses were performed using JMP Pro 14 software (SAS Institute, Cary, NC, USA).

## 3. Results

The patients’ demographic data are shown in Table 1. DVT was detected in 26 patients (5.2%) before the index THA, all of which were asymptomatic. Proximal DVT was found in nine cases (six in the femoral vein and three in the popliteal vein) and distal DVT (in calf veins) was found in 17 cases. When DVT was found, the surgeon consulted with cardiologists and the cardiologists decided whether an anticoagulant should be administered for each case. For the patients who had proximal DVT, an anticoagulant was newly started before the index surgery in four cases. No anticoagulant was used before surgery in two cases. The remaining three cases had been taking anticoagulants before the ultrasound screening and they continued the drug after the detection of DVT. For the patients with distal DVT, an anticoagulant was newly administered before THA in seven cases. No anticoagulant was used before THA in eight cases. The remaining two cases continued taking the anticoagulant they had been taking before the screening. The average age in the DVT group was significantly higher than that in the no-DVT group (70.5 vs. 64.5 years, *p* = 0.0083). The DVT and no-DVT groups contained 24 (92.3%) and 385 (81.2%) women, respectively (*p* = 0.12). The mean BMI was 23.6 ± 4.0 kg/m^2^ (range, 18.2–32.2 kg/m^2^) in the DVT group and 24.3 ± 4.2 kg/m^2^ (range, 12.2–41.8 kg/m^2^) in the no-DVT group (*p* = 0.63). Overall, 54 patients were currently using corticosteroids. The mean daily dose among these 54 patients was 7.5 ± 4.9 mg (range, 1–20 mg). Ten patients (38.5%) in the DVT group, but only 44 (9.3%) in the non-DVT group, were currently taking steroids; the proportion was significantly higher in the DVT group (*p* < 0.0001). Twenty patients (76.9%) in the DVT group and 327 (69.0%) in the no-DVT group had a history of major surgery (*p* = 0.22). When the history of major surgery was limited to 12 months prior to the index THA, the difference between the two groups was more marked (26.9% vs. 13.3%). More patients in the DVT group tended to have a history of major surgery within the last 12 months prior to the index THA, although the difference was not significant (*p* = 0.053). Significantly more patients had a history of DVT in the DVT group than in the no-DVT group (23.1% vs. 1.1%, *p* < 0.0001). Among the comorbidities, collagen disease was present in more patients in the DVT group than in the no-DVT group (*p* = 0.023). 

The breakdown of collagen diseases in each group is shown in Table 2. Significantly more patients were under the administration of anticoagulants in the DVT group than in the no-DVT group (23.1% vs. 9.5%). There was no difference in the incidence of DM, Af, CHF, or malignant disease between the DVT and no-DVT groups. The percentage of current smokers was not significantly different between the DVT and no-DVT group.

The clinical scores are shown in Table 3. Hereafter, the score and scale are given as the mean ± standard deviation (range). The mean UCLA activity score was 2.64 ± 0.91 (2–5) in the DVT group and 3.75 ± 1.33 (1–8) in the no-DVT group (*p* < 0.0001). The mean OHS was 22.50 ± 12.32 (1–45) in the DVT group and 29.73 ± 9.97 (1–48) in the no-DVT group (*p* = 0.006). The VAS score was higher in the DVT group (6.88 ± 2.50 (0–10)) than in the no-DVT group (6.04 ± 2.82 (0–10)); however, the difference was not significant (*p* = 0.21). The difference in the HHS between the DVT group (50.9 ± 17.79 (17.0–84.1)) and no-DVT group (52.66 ± 16.04 (5.7–99.6)) was also not statistically significant (*p* = 0.55). 

Because hip clinical scores reportedly have strong correlations with one another [24,25,26,27,28,29], the correlation between each combination of the four clinical measurements (UCLA activity score, OHS, HHS, and VAS score) was tested using Spearman’s rank correlation coefficient. The *p*-value and Spearman’s rank correlation coefficient for each combination are shown in Table 4 and Table 5, respectively. All six combinations of the four clinical measurements (UCLA activity score, OHS, HHS, and VAS score) had a statistically significant correlation. All combinations except the UCLA activity score and VAS score (coefficient = −0.14) had an absolute coefficient value of 0.3 to 0.6, which is considered a moderate correlation. 

A multivariate logistic regression analysis was performed with two different models: regression with parameters reaching a significance of *p* < 0.05 in the univariate analysis (i) with the OHS as the representative clinical measurement and (ii) with the UCLA activity score as the representative clinical measurement. This was performed to avoid potential multicollinearity between the OHS and UCLA activity score, which was implied in Table 4 and Table 5 and shown in previous literature [24], demonstrating a significant correlation between the OHS and UCLA activity score (r = 0.48, *p* < 0.0001).

In Model 1, age, a history of DVT, current steroid use, and the OHS were significantly correlated with preoperative DVT (Table 6). In Model 2, age, a history of DVT, current steroid use, and the UCLA activity score were significant risk factors for preoperative DVT (Table 7). Age, a history of DVT, and current steroid use were significant risk factors for preoperative DVT in both models, and the UCLA activity score and OHS were significantly correlated with preoperative DVT in each model when involved as an independent variable. 

The mean UCLA activity score in patients with proximal DVT was 2.5 ± 0.5, whereas that in patients with distal DVT was 2.7 ± 1.0. The difference was not significant (*p* = 0.69). There was no significant difference between the proximal DVT group and distal DVT group in HHS (46.8 ± 12.7 vs. 53.5 ± 20.2, *p* = 0.35), or in OHS (21.9 ± 9.0 vs. 22.9 ± 14.2, *p* = 0.78). 

**Figure 1:**
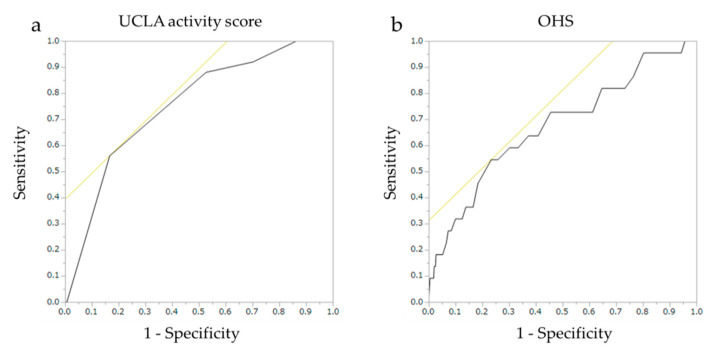
The ROC curves of the (**a**) UCLA activity score and (**b**) OHS. ROC, receiver operating characteristic; UCLA, University of California Los Angeles; OHS, Oxford Hip Score.

The ROC curves are shown in Figure 1. The AUCs (95% confidence interval) for the UCLA activity score and OHS were 0.755 (0.645–0.840) and 0.673 (0.536–0.786), respectively. The optimal predictive cutoff for the UCLA activity score was 2 (sensitivity, 56.0%; specificity, 83.3%). Its positive (PPV) and negative (NPV) predictive values were 15.7% and 94.7%, respectively. Its Youden Index was 0.393. The optimal predictive cutoff for OHS was 22 (sensitivity, 54.5%; specificity, 76.6%; PPV, 10.4%; NPV, 97.1%). Its Youden Index was 0.312 (Table 8).

## 4. Discussion

In the present study, 5.2% of the patients who were scheduled for THA had DVT, even before the surgery. This proportion is in good accordance with those of previous studies (5.8%–12.3%) [4,5]. A few reports have focused on preoperative DVT before other types of surgery. Akeda et al. [31] reported that preoperative DVT was identified using a Doppler ultrasound in nine (4.3%) of 209 patients undergoing spinal surgery. In another study, preoperative DVT was identified using a Doppler ultrasound in 25 (3.8%) of 654 patients undergoing surgery for gynecologic malignancy [32].

Older age is a predictor of preoperative DVT after THA [6,7,8,9,10,11,12,13] and before THA [5]. Our results also demonstrated that older patients were significantly more likely to have DVT before THA. 

Although a high BMI of >30 kg/m^2^ has been associated with a higher risk of DVT after THA [6,17] and PTE after THA [33], we found no correlation between BMI and preoperative DVT. Likewise, two other studies also showed no significant correlation between a high BMI and preoperative DVT [4,5]. Because the average BMI in the present study and the two above-mentioned studies was relatively low (23–24 kg/m^2^), a possible explanation is that the studies contained too few patients with obesity to examine the effect of excess body weight on the incidence of DVT. A larger number of patients with obesity should be evaluated to more fully examine the effect of a high BMI.

Corticosteroid use is reportedly associated with DVT after surgery [34,35,36]. In the present study, corticosteroid use significantly increased the risk of DVT, even before surgery.

The effect of DM on the risk for DVT after THA has been discussed in several studies. In their meta-analysis of five studies, Zhang et al. [37] showed no correlation between DM and postoperative DVT (odds ratio (OR): 1.02, *p* = 0.88). We also found no significant correlation between a history of DM and the risk of DVT before THA.

Some authors have referred to malignancy as a significant risk factor for postoperative DVT after THA [6,7,11], whereas others have shown no significant difference between malignancy and DVT, providing an OR of 1.00 (0.72–1.39) [10] and 1.00 (0.32–3.11) [16]. In the present study, a history of malignancy was not associated with preoperative DVT; however, we could not divide patients with active and inactive malignancies because it was difficult to determine the exact stage of the tumor or the recurrence only from the medical records in our institute.

Patients with a history of DVT have a higher risk of postoperative DVT after THA [6,9,10]. The present study also indicated that a history of DVT was significantly correlated with an increased risk of preoperative DVT before THA. Although no previous studies have demonstrated an increased risk of preoperative DVT prior to THA for patients with a known history of DVT, a history of DVT would understandably be a predictor of preoperative DVT. 

Some studies have shown that CHF was associated with an increased risk of postoperative DVT after arthroplasty [9,11,13], whereas Beksac et al. [6] reported no correlation between CHF and postoperative DVT. In the present study, CHF was not a predictor of preoperative DVT. However, whether Af is associated with the risk of postoperative DVT has not been rigorously studied. Previous studies that did not focus on the postoperative period demonstrated that Af was associated with an increased risk of DVT [38,39]. Our results showed no association between Af and preoperative DVT. However, only 16 patients had CHF and 15 had Af. It would be difficult to draw a conclusion regarding CHF and Af as risk factors for preoperative DVT in this cohort. 

Conclusions regarding the effect of anticoagulants on the risk of preoperative DVT would also be difficult to draw in this study. While anticoagulant use was associated with an increased risk of DVT in the univariate model, the association was not significant in the multivariate models. This was unexpected because anticoagulants themselves are expected to prevent DVT. Most patients with a history of DVT might have been taking anticoagulants to prevent the recurrence or expansion of DVT, which could have strongly affected the association between anticoagulant use and DVT risk in this study.

A history of major surgery is also a reported risk factor for DVT [4,30]. In this study, we included a history of major surgery itself and major surgery within the last 12 months, because many patients had undergone major surgeries a decade previously or could not recall exactly when the previous major surgery was performed. As a result, a history of major surgery itself was not a risk factor, but major surgery within the last 12 months tended to increase the risk of preoperative DVT (*p* = 0.053). 

Hip clinical scores reportedly have strong correlations with one another [24,25,26,27,28,29]. Naal et al. [24] showed a significant correlation between the UCLA activity score and OHS (r = 0.48, *p* < 0.0001) and between the UCLA activity score and HHS (r = 0.56, *p* < 0.0001) among patients undergoing THA. Beaulé et al. [27] found a strong correlation between the UCLA activity score and HHS with a Pearson correlation coefficient of 0.57. Sechriest et al. [28] found that the UCLA score was positively correlated with the HHS (r = 0.52, *p* = 0.001). Parsons et al. [26] reported that the OHS was correlated with the HHS in their hip resurfacing series. Taking these correlations into account, we considered that if the clinical scores were employed in the same multivariate regression, there must be a significant risk for multicollinearity that should be avoided to strictly examine the effect of each hip score on the risk of preoperative DVT. Therefore, we performed separate multivariate logistic regression analyses using either the UCLA activity score or OHS as the representative clinical score, whereas in the univariate analysis, both the UCLA activity score and OHS showed a significant correlation with the incidence of preoperative DVT. The effect of clinical scores on the location of the thrombosis (proximal or distal) was also examined; however, there was no association between those scores (UCLA activity score, OHS, or HHS) and the location of thrombosis. 

Collagen disease and current steroid use might also be confounding factors that are significantly correlated with each other. However, because their variance inflation factors were <2, both of these parameters were included in each multivariate model. While steroid use was significantly associated with the presence of preoperative DVT, collagen disease was not an independent risk factor for preoperative DVT. 

This study had some limitations. First, the number of patients with preoperative DVT was relatively small. If more patients had preoperative DVT, various other potential confounders could be included in the analysis. Second, this cohort included various types of collagen diseases. Although each disease might have had a different effect on the tendency to cause DVT, these diseases were handled as a single disease group because of the small number of patients.

In conclusion, older age, current corticosteroid use, a history of DVT, a lower UCLA activity score, and a lower OHS were significant risk factors for preoperative DVT in patients scheduled for THA. The commonly used activity and hip function scales could help predict the risk of preoperative DVT.

## Figures and Tables

**Table 1:** Demographics of each group.

Variables		Overall	DVT	No DVT	*p* Value ^#^
*n*		500	26	474	
Sex	Male	91	2	89	0.12
	Female	409	24	385	
Age, years		64.79 ± 12.7 (22–89)	70.0 ± 14.6 (35–87)	64.5 ± 12.5 (22–89)	0.0083
BMI, kg/m^2^		24.0 ± 4.0 (12.2–41.7)	23.6 ± 3.9 (18.2–32.2)	24.3 ± 4.2 (12.2–41.8)	0.63
Current steroid use		54	10/26	44/474	<0.0001
Previous DVT history		11/500	6/26	5/474	<0.0001
Current anticoagulant use		51/500	6/26	45/474	0.026
Atrial fibrillation		15/500	1/26	14/474	0.80
Congestive heart failure		16/500	1/26	15/474	0.85
Major surgery		347/500	20/26	327/474	0.22
Major surgery within last 12 months		70/500	7/26	63/474	0.053
Diabetes mellitus		54/500	3/26	51/474	0.9
Collagen disease		55/500	7/26	48/474	0.023
Malignancy		78/500	3/26	75/474	0.55
Current smoking		36/500	1/26	35/474/	0.50

Data are shown as the number of patients or the mean ± standard deviation (range). DVT: deep venous thrombosis and BMI: body mass index. ^#^ Comparison of the presence and absence of preoperative DVT.

**Table 2:** Breakdown of collagen disease in each group.

Type of Collagen Disease	Overall (*n* = 500)	DVT (*n* = 26)	No DVT (*n* = 474)
Rheumatoid arthritis	23	2	21
Systemic lupus erythematosus	15	2	13
Dermatomyositis	8	1	7
Systemic sclerosis	3	3	0
Sjögren syndrome	3	0	3
Polymyalgia rheumatica	2	0	2
Polyarteritis nodosa	1	0	1
Adult Still’s disease	1	0	1
Behçet’s disease	1	0	1
Eosinophilic granulomatosis with polyangiitis	1	0	1

DVT: deep venous thrombosis.

**Table 3:** Clinical measurement scores for each group.

Variables	Overall	DVT	No DVT	*p* Value ^#^
Oxford Hip Score	29.4 ± 10.2	22.5 ± 12.3	29.7 ± 10.0	0.0060
Harris Hip Score	52.6 ± 16.1	50.9 ± 17.8	52.7 ± 16.0	0.55
UCLA activity score	3.69 ± 1.34	2.64 ± 0.91	3.75 ± 1.33	<0.0001
VAS	6.08 ± 2.81	6.88 ± 2.50	6.04 ± 2.82	0.21

DVT: deep venous thrombosis, UCLA: University of California Los Angeles, and VAS: visual analog scale. ^#^ Comparison of the presence and absence of preoperative DVT.

**Table 4:** Probability (*p*) value for the correlation of each combination of clinical measurements.

	UCLA	OHS	HHS	VAS
UCLA	<0.0001	<0.0001	<0.0001	0.0039
OHS		<0.0001	<0.0001	<0.0001
HHS			<0.0001	0.0032
VAS				<0.0001

OHS: Oxford Hip Score, HHS: Harris Hip Score, UCLA: University of California Los Angeles, and VAS: visual analog scale.

**Table 5:** Spearman rank correlation coefficient for each combination of clinical measurements.

	UCLA	OHS	HHS	VAS
UCLA	1.0	0.34	0.43	−0.14
OHS		1.0	0.42	−0.60
HHS			1.0	−0.31
VAS				1.0

OHS: Oxford Hip Score, HHS: Harris Hip Score, UCLA: University of California Los Angeles, and VAS: visual analog scale.

**Table 6:** Multivariate logistic regression analysis using two parameters reaching *p* < 0.05 in univariate testing with OHS as a representative clinical measurement.

Variables	Odds Ratio	95% CI	*p* Value	VIF
OHS	0.95	0.91–0.99	0.031	1.02
Age	1.07	1.03–1.12	0.00056	1.12
Previous history of DVT	27.15	3.31–222.53	0.0021	1.18
Current anticoagulant use	0.48	0.09–2.63	0.39	1.20
Collagen disease	0.93	0.19–4.64	0.93	1.60
Current steroid use	10.45	2.31–47.34	0.0023	1.67

OHS: Oxford Hip Score, DVT: deep venous thrombosis, CI: confidence interval, and VIF: variance inflation factor.

**Table 7:** Multivariate logistic regression analysis using two parameters reaching *p* < 0.05 in univariate testing with the UCLA activity score as a representative clinical measurement.

Variables	Odds Ratio	95% CI	*p* Value	VIF
UCLA activity score	0.49	0.28–0.84	0.0099	1.16
Age	1.07	1.02–1.12	0.0027	1.27
Previous history of DVT	74.98	8.54–658.18	<0.0001	1.17
Current anticoagulant use	0.34	0.056–2.06	0.24	1.20
Collagen disease	1.03	0.23–4.68	0.97	1.55
Current steroid use	9.32	2.14–40.68	0.0030	1.69

UCLA: University of California Los Angeles, DVT: deep venous thrombosis, CI: confidence interval, and VIF: variance inflation factor.

**Table 8:** The diagnostic value of the UCLA activity score and OHS.

Variables	AUC	Youden Index	Predictive Cutoff	Sensitivity	Specificity	PPV (%)	NPV (%)
UCLA activity score	0.755	0.393	2	56.0	83.3	15.7	94.7
OHS	0.673	0.312	22	54.5	76.6	10.4	97.1

UCLA: University of California Los Angeles, OHS: Oxford Hip Score, AUC: area under the curve, PPV: positive predictive value, and NPV: negative predictive value.
